# The Relationship between Parental Depressive Symptoms, Family Type, and Adolescent Functioning

**DOI:** 10.1371/journal.pone.0080699

**Published:** 2013-11-18

**Authors:** Dominik Sebstian Sieh, Johanna Maria Augusta Visser-Meily, Anne Marie Meijer

**Affiliations:** 1 Research Institute of Child Development and Education, University of Amsterdam, The Netherlands; 2 Center of Excellence for Rehabilitation Medicine, Rehabilitation Center De Hoogstraat, Utrecht, The Netherlands; Chiba University Center for Forensic Mental Health, Japan

## Abstract

It is evident that parental depressive symptoms negatively influence adolescent behavior and various psychosocial outcomes. Certain family types like families with a chronically ill parent and single parent families are more vulnerable to parental depressive symptoms. However, the relationship between these symptoms, family type, and adolescent functioning remains largely unclear. This study examined relations between self-report of parental depressive symptoms and adolescent functioning in 86 two-parent families including a parent with a chronic medical condition, 94 families with healthy single parents, and 69 families with 2 healthy parents (comparison group). Parents completed the Beck Depression Inventory. Adolescents filled in the Youth Self-Report measuring problem behavior, and other instruments measuring psychosocial outcomes (stress, grade point average, school problems, and self-esteem). Multilevel analyses were used to examine the effects of family type, parental depressive symptoms, adolescents' gender and age, and interaction effects on adolescent functioning. The results indicated that adolescents with chronically ill and single parents had a lower grade point average (*p*<.01) than the comparison group. Adolescents of single parents reported more internalizing problems (*p*<.01) and externalizing problems (*p*<.05) than children from the other family types. Parental depressive symptoms were strongly related to child report of stress (*p*<.001). Adolescents of depressed chronically ill parents were particularly vulnerable to internalizing problems (interaction effect, *p*<.05). Older children and girls, and especially older girls, displayed more internalizing problems and stress. It can be concluded that growing up with a chronically ill parent in a family with 2 parents may have less impact on adolescent problem behavior than growing up in a single parent family. Health practitioners are encouraged to be attentive to the unique and combined influence of family type and parental depressive symptoms on adolescent functioning. Older and female adolescents deserve particular attention.

## Introduction

Both maternal and paternal depressive symptoms are associated with negative consequences for children's development, including internalizing and externalizing problems, and general psychopathology [1]–[3]. Externalizing problems prove to be equally related to depression in fathers and mothers, whereas internalizing problems seem more closely related to depression in mothers. All associations are small in magnitude, but Connell and Goodman [1] found substantial variability across studies, highlighting the need to control for theory-based moderators, such as children's gender and age. Goodman et al. [2] stress the need to identify subgroups of children who are at greater risk for problem behavior. Subgroups of at-risk children may be defined by the presence of elevated depressive symptoms in parents.

**Figure 1:**
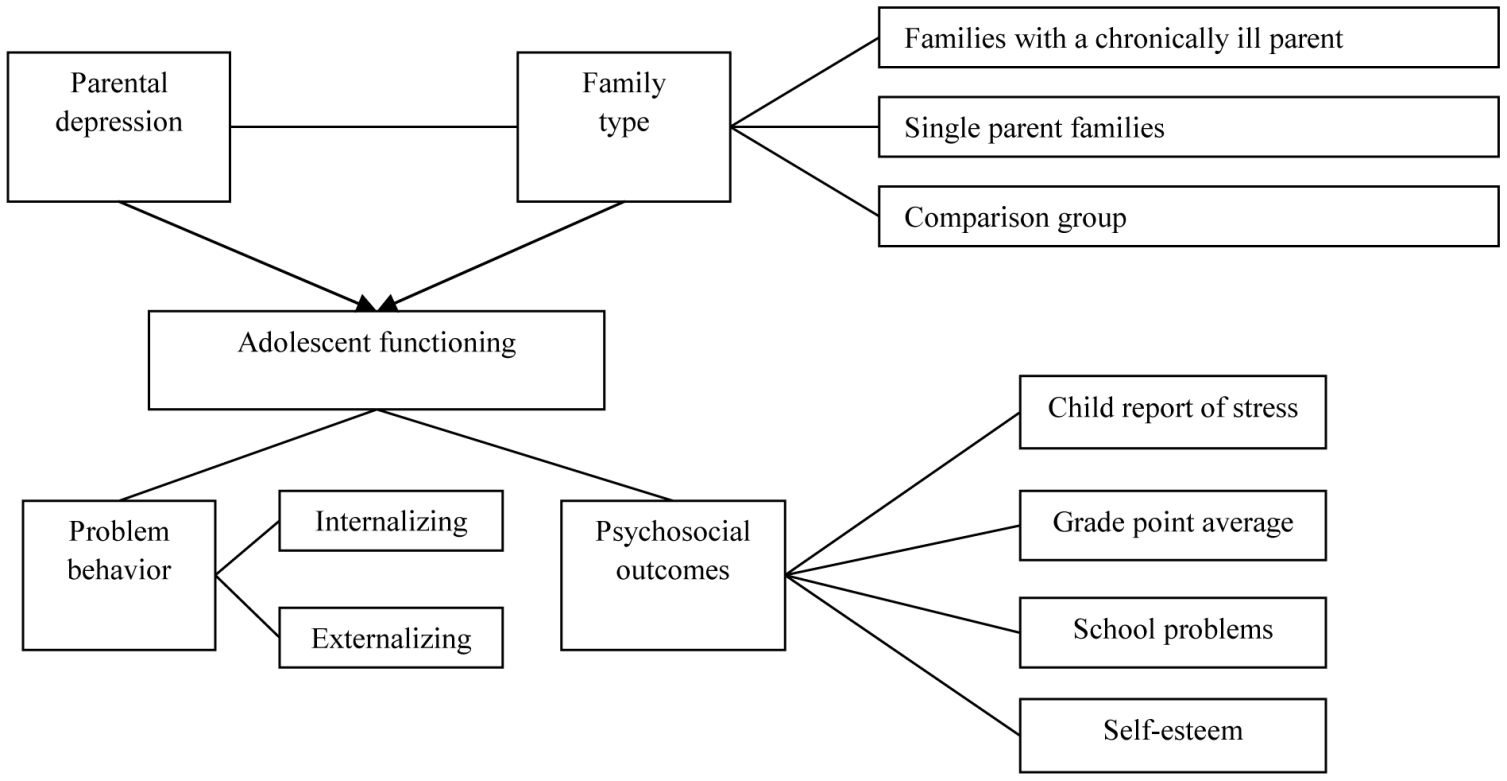
Parental depressive symptoms and family type as predictors for adolescent outcomes.

Several studies revealed that two family types prove to be at increased risk for parental depression. First, families including a parent diagnosed with a chronic medical condition (CMC) show more depressive symptoms in patients and spouses [4]–[6]. Second, parental depressive symptoms appear to be more elevated in single parents than in parents from two-parent families [7]–[9]. There is evidence that children of these family types (target groups) have worse outcomes than children of two-parent families with healthy parents on a number of measures. The most prominent explanation is that the target groups are confronted with lower physical and emotional availability of at least one of the main caregivers [10]–[12]. Specifically, children with parental CMC show more internalizing problem behavior [5], more stress [13], [14], and lower overall academic functioning than other children [15]. Similarly, children from single parent families manifest an increased risk for internalizing problems, externalizing problems (delinquent behavior and vandalism), and they display comparatively low academic functioning and self-esteem [16], [17]–[23]. Figure 1 illustrates a model explaining adolescent functioning as a result of the specific and combined effect of family type and parental depressive symptoms.

The question arises as to whether children of parents with CMC and children of single parents may function worse as a result of increased parental depressive symptoms that are related to family type. Besides, it needs to be clarified whether family type and elevated depressive symptoms in parents synergistically potentiate the effect on adolescent functioning. To date, there are no studies answering these questions. Aiming to elucidate this complex matter, our study addresses a few shortcomings of prior research. To begin, most studies have examined children in families with parental CMC and children in single parent families without controlling for parental depressive symptoms [5], [7], [9], [16], [24], hindering distinct conclusions [2]. Research to date has mainly focused on child functioning in association with either parental depressive symptoms or family type. On top of that, there are methodological shortcomings that interfere with drawing firm conclusions. Exclusive use of parent report of child functioning may affect results due to biased reporting from depressed parents [25]. Interestingly, even non-depressed parents seem to evaluate deviant behavior in their children as more problematic than children themselves [26]. Further, the impact of paternal depressive symptoms on child functioning has been neglected [1], which may inherently result in overemphasizing of maternal depressive symptoms. Finally, beyond family type, also a family cluster effect, that is, the effect of belonging to the same family, may play a role [16]. Members of the same family are more similar to each other than members of different families, resulting in dependence of observations. Unlike prior research, this study statistically controls for the family cluster effect, thereby avoiding that the significance of potential effects is overestimated. Accordingly, it can be calculated how much variance in adolescent scores is explained by differences between families.

Consequently, the aim of this study is to investigate the unique and combined influence of family type and parental depressive symptoms on adolescent functioning. We examine main effects and interaction effects between family type, parental depressive symptoms, and children's gender and age to detect potentiated effects. By choosing families affected by parental CMC and single parent families (target groups), and two-parent families without parental CMC (comparison group), we increase insight into key determinants of family and adolescent functioning, that is, family type and parental mental health. To explore a broad range of adolescent functioning, we examine both problem behavior and other psychosocial outcomes (child report of stress, grade point average, school problems, and school-related self-esteem). In our study, we use the mean of maternal and paternal depressive symptoms scores to take the influence of both main caregiver and alternative caregiver into account, tackling the level of overall parental depressive symptoms in the family. Another rationale for using dyadic scores is to use all data for single parent families instead of selective data of single mothers or single fathers only. However, as research suggests that maternal depressive symptoms have more impact on child functioning than paternal depressive symptoms [1], we repeat our analyses using maternal depressive scores to explore the effect of maternal depressive symptoms only.

The empirical basis [6], [8] leads to the assumption that parents of families affected by parental CMC and single parents score higher on overall depressive symptoms than parents from families with two healthy parents, and consequently, adolescents growing up in these family types may manifest more internalizing and externalizing problem behavior than adolescents from healthy families [5], [22]. Besides, more stress, a lower grade point average, more school problems, and lower school-related self-esteem are expected in the target groups.

The questions our study responds to, makes up for high clinical relevance. To illustrate, health professionals may benefit from knowledge about the relationship between parental depressive symptoms, family type, and adolescent functioning, because this knowledge helps to evaluate the risk for poor adolescent functioning and may help health professionals to make a decision of which children should be screened or targeted for interventions. For instance, the knowledge that a certain family type combined with a high parental depression score constitutes a risk factor for poor adolescent functioning can contribute to alertness among professionals who then may consider preventive interventions for these children and the whole family. This research is essential because three very common issues with potentially high implications for adolescent development, that is parental chronic medical condition, single parenthood, and parental depressive symptoms, are combined into one study to examine how they may challenge adolescent problem behavior and psychosocial outcomes.

## Methods

### Ethics statement

This study has been approved by the ethics committee of the Research Institute of Child Development and Education of the University of Amsterdam. Participants provided their written informed consent to participate in this study. We also obtained written informed consent from the parents on the behalf of the minors/children involved in our study. The ethics committee approved the consent procedure.

### Participants

We included children aged 10 to 20 years living at home with their parents. Families were recruited through rehabilitation and community centers, schools, hospitals, and general practitioners' offices across the Netherlands. All family members completed a test battery at home under assistance of research assistants who had been trained according to a protocol. A wide age range was chosen to reach a large number of children during adolescence. Children of two-parent families, in which either one or two parent(s) had an impairing medical condition lasting longer than 6 months, composed families with a chronically ill parent. Only those participants were included that had a CMC associated with functional impairment, that is, problems in self-care, mobility, or psychosocial functioning. Cancer was excluded because it is not a chronic condition per definition; it can be cured or it can be lethal. Also, a meta-analysis found that problem behavior in children is less pronounced in cancer studies than in non-cancer studies [5]. Children of single parent families had to live with one parent most of the week, while no other (step)parent was present in the household. Children from the comparison group had two parents without CMC. Exclusion criteria for adolescents were insufficient command of Dutch, residency outside of the Netherlands, severe somatic diseases, and cognitive disabilities. Having a light somatic disease like asthma was not an exclusion criterion for participation of adolescents.

### Measurements

Psychometric properties (number of items, range, and reliability) of all instruments are shown in Table 1.

**Table 1:** Psychometric Properties.

	Items	Range	Internal consistency (Cronbach's alpha)		
	(*n*)		families with a chronically ill parent	single parent families	comparison group
Internalizing problems (YSR)	31	0–62	.91	.88	.78
Externalizing problems (YSR)	30	0–60	.81	.70	.78
Stress (SVK)	17	17–68	.88	.83	.80
School problems	11	0–22	.75	.78	.65
School-related self-esteem (SPQ)	8	8–40	.90	.85	.88
Parental depressive symptoms (BDI)	21	0–42	.85	.82	.79

*Note.* YSR =  Youth Self Report; SVK =  Dutch Stress Questionnaire; SPQ =  School Perception Questionnaire; BDI =  Beck Depression Inventory.

#### Adolescent problem behaviour

Internalizing and externalizing problems were measured with the Youth Self-Report (YSR) from Achenbach [27]. Items were summed to obtain a total score for internalizing symptoms (i.e., anxious/depressed behavior, withdrawn/depressed behavior, and somatic complaints) and externalizing symptoms (i.e., aggressive and rule-breaking behavior). Answers could be rated as *not true* (0), *somewhat/sometimes true* (1) or *very/often true* (2). We only used raw scores because they are more specific than T-scores and because we controlled for children's gender and age in the analyses.

#### Child report of stress

Child report of stress was determined with the Dutch Stress Questionnaire for Children [28], using a scale from 1 (*completely not true for me*) to 4 (*completely true for me*). Higher scores represent a higher perception of psychological stress. An exemplary item was “*I often feel rushed*”. This questionnaire has been widely used in the Netherlands to measure stress in children, showing high reliability [29]–[31].

#### Adolescent academic functioning

Academic functioning referred to adolescents' report of grade point average (GPA), school problems, and school-related self-esteem. According to the Dutch scholar system, children indicated the average GPA for all assignments from the previous school year on a scale from 4 and below (*insufficient*) to 10 (*excellent*). School problems were assessed with an additional subscale with the YSR format, for instance “*I have been sent out of class for misbehavior*” [32], [33]. Further, we used the School Perception Questionnaire to give an indication of the school-related self-esteem on a 4-point scale from 0 (*completely true*) to 4 (*completely not true*), for example “*I think that I perform well at school*”. The School Perception is a valid measure [34]. Higher scores mean higher self-esteem. Lastly, we asked adolescents if they had ever failed a school year.

#### Parental depressive symptoms

Parental depressive symptoms were measured with the Beck Depression Inventory (BDI), with 21-items including affective, cognitive, behavioral, and somatic signs of depression. The BDI has a 3-point scale with a maximum score of 42, indicating severe depression [35]. On behalf of the data analysis, for families with a chronically ill parent and the comparison group, we calculated mean depression scores by dividing the sum of mothers' and fathers' scores by 2. Exploratively, we also used the depression scores of mothers. Fathers' depression scores were not considered because only 20% of the single parents were fathers.

### Procedure

Participants of all family types were recruited across the Netherlands in schools, community centers, general health practitioners' offices, and public libraries via postings and letters. Participants of families with a chronically ill parent were additionally recruited in rehabilitation centers and hospitals. Besides, information was posted on websites of the major national patient organizations (e.g., Dutch association of Parkinson disease). After written informed consent had been given, research assistants made an appointment to administer questionnaires at the families' homes. Adolescent participants received a gift voucher, a cinema ticket, or a mobile phone cover after completion of the questionnaires.

### Data analysis

Linear Mixed Modeling (LMM) for continuous outcome variables with maximum likelihood (ML) estimation was used to examine the effect of family type, adolescents' gender and age, parental depressive symptoms, and interaction effects on the outcome variables [36]. Linear mixed modeling corrects for the family cluster effect, counteracting the violation of independent observations. This clustering effect refers to the fact that children from the same family are more similar to each other than children from different families. We produced dummies for family type, meaning that we used a variable that took the value 0 or 1 to indicate the absence or presence of the variable in question. In this way we compared the data from children with a chronically ill parent or single parent (target groups  = 1), respectively, with the data of the remaining families (controls  = 0) for each outcome variable separately. To begin, we conducted descriptive statistics, using LMM. We used the Bonferroni test to examine which family types differed from each other, responding to the issue of multiple comparisons.

In the first step of model fitting, an intercept-only model was used to identify the family cluster effect for the outcome variables. We calculated the Intra Class Correlations (ICC's), giving an indication of how much variance in adolescent scores was explained by differences between families [37]. In the second step, we used a conditional model to test the fixed or between-subjects effects of family type on adolescent outcomes. Third, in addition to family type, we entered adolescents' gender and age, and parental depressive symptoms into the model. In the first three steps, the individual variables were tested on significance taking into account the divided variance with the other variables that were entered. In these cases, a significant effect of a variable referred to a main effect. In the fourth step, we tested all possible interaction effects between family type, adolescents' gender and age, and parental depressive symptoms. For example, by the interaction effect of adolescent gender by parental depressive symptoms we aimed to test whether the effect of parental depressive symptoms on adolescent outcomes was larger for girls than for boys. The fourth step yielded the best fitting model including interaction effects, in which only significant effects (*p*<.05) were accepted. Non-significant effects were removed step-by-step depending on the level of significance. When an interaction effect was significant, the corresponding variables for main effects were maintained for the sake of interpretability, irrespective of whether these effects were significant.

Finally, we examined differences between adolescents of families with parental CMC and single parent families separately, using multi level modeling. To explore whether maternal depressive symptoms might have more effect on adolescent functioning than dyadic scores, we only used maternal depressive symptom scores instead of dyadic scores and re-ran the analyses.

For comparability of the estimates of regression analyses, we standardized all independent variables. Intercepts in fixed models represent the mean score of participants at baseline under the assumption that the included independent variables are equal to zero. Regression coefficients represent the differential effects of independent variables on the outcome variables. As the regression coefficient is significant, one unit increase/decrease of the independent variable is expected to result in one unit increase/decrease of the outcome variable. Positive coefficients for child's gender refer to girls. The analyses were conducted using IBM SPSS statistics package, version 20.0. The significance level for all tests was *p*<.05, two-tailed.

## Results

### Descriptive statistics

This study presents the data of 389 adolescents and their parents from 3 family types. Families with a chronically ill parent included 86 two-parent families with 140 children and 172 parents with one or two parent(s) affected by an impairing CMC. Single parent families consisted of 94 single parent families with 135 children. The comparison group was composed by 69 two-parent families with 114 children and 138 parents without CMC. Descriptive statistics per family type are shown in Table 2. In all family types, 52% of the children were girls and children were between 10 and 20 years of age (mean age  =  approximately 15.0 years).

**Table 2:** Descriptive Statistics for Families with Chronically Ill, Single, and Healthy Parents.

	Families with a chronically ill parent	Single parent families	Comparison group	*p-*value
Number of families	86	94	69	
Average number of children per family	1.63	1.96	1.65	
*Children (n)*	140	135	114	
gender (female)	52.86%	52.59%	52.63%	
mean age (*SD*)	15.15 (2.32)	15.43 (2.70)	14.97 (2.25)	
mean educational level^1^ (*SD*)	6.81 (3.23)	6.84 (3.31)	7.19 (2.93)	
failed at least one school year	16.43%	20.70%	19.15%	
mean internalizing problems (*SD*)	9.68 (8.54)	10.44 (7.86)	7.37 (5.04)	**
mean externalizing problems (*SD*)	7.24 (5.42)	8.64 (4.78)	7.28 (5.03)	
mean stress (*SD*)	34.71 (8.13)	35.24 (7.36)	33.01 (6.11)	*
mean grade point average^2^	6.94 (.85)	6.95 (.75)	7.26 (.76)	**
mean school problems (*SD*)	2.96 (3.00)	3.57 (3.27)	2.77 (2.49)	*
mean school-related self-esteem (*SD*)	32.13 (5.70)	31.88 (5.11)	33.30 (4.87)	*
*Parents (n)*	172	94	138	
mean age (*SD*)	46.64 (5.66)	47.35 (5.58)	47.79 (5.08)	
mean educational level^3^ (*SD*)	4.12 (1.33)	4.27 (1.25)	4.33 (1.25)	
monthly income in Euro's (*SD*)	2910 (885)	2060 (943)	3190 (870)	***
mean depressive symptoms (*SD*)	9.54 (7.49)	5.53 (5.36)	4.11 (3.94)	***
no depression^4^	65%	85%	91%	-
mild depression	20%	14%	8%	-
moderate depression	8%	0%	0%	-
severe depression	7%	1%	1%	-

*Note.*

1 School or education level ranges from 1 =  elementary school to 12 =  university.

2 Grade point average ranges from 4 and below (insufficient) to 10 (excellent).

3 Education level ranges from 1 =  elementary school to 6 =  university.

4 Depression scores are divided into categories ranging from 0 to 10 (not depressed), 11 to 17 (mildly depressed), 18 to 23 (moderately depressed), and 24 to 42 (severely depressed).

*p<.05.

**p<.01.

***p<.001. All significance tests are based on linear mixed modeling except for parental depressive symptoms which was conducted using ANOVA.

In families with a chronically ill parent, parental CMC concerned the mother in 63% of cases and included multiple sclerosis (28.1%), rheumatoid arthritis (20.2%; e.g., Bechterew's disease), neuromuscular disease (16.9%; e.g., hereditary motor and sensory neuropathy), traumatic brain injury (14.6%), spinal cord injury (7.9%), Parkinson disease (5.6%), and inflammatory bowel disease (5.6%; e.g. Crohn's disease) or diabetes type I with physical complications (1.1%). The mean time since diagnosis was 11.8 years, ranging from 7 months to 49 years. Single parent families were mainly composed by single mothers (80.6%).

Adolescents of the three family types did not differ in gender, age, educational level, or the percentage of failed school years, see Table 2. Only 9% of the children were below 11 years of age or older than 18 years. Parents' age and educational level did not differ between the family types (*p*>.10). All three groups were mostly composed of highly educated parents and adolescents. Almost all of the participants in the three groups were of European descent with a Western cultural influence. One child with a chronically ill parent was from Suriname. In single parent families, two children were from Curaçao, one child from the United States and one child from Colombia. In the comparison group, two children were from Suriname, one child from Indonesia and one child from Yemen. The monthly family income significantly differed between the three family types, *F*(2,310) = 45.06, *p*<.001. The Bonferroni test showed that families with two healthy parents had more income than single parent families (mean difference  = 1129 €, *p*<.001). Families with parental CMC had more income than families with single parents (mean difference  = 859 €, *p*<.001). Parental depressive symptoms also differed between the family types, *F*(2,390) = 34.08, *p*<.001. Parents in families with parental CMC were more depressed than parents of the other family types (*p*<.01). Single parents and parents from the comparison group did not differ in depressive symptoms scores. Based on the norms of the BDI, clinical levels of depressive symptoms were more common in parents with CMC (see Table 2).

### Empty model

The ICC's for children's internalizing problems, child report of stress, and grade point average (GPA) were significant and varied between ρ = .18 and ρ = .28, showing that adolescents within the same family scored similarly on these outcome variables. The ICC for the remaining outcome measures was not significant (*p*>.05), meaning that a high percentage of individual characteristics determined externalizing problems, school problems, and school-related self-esteem, see Table 3. Overall, the family cluster effect was strong enough to justify multilevel analysis.

**Table 3:** Random Effects in the Empty Model and Fixed Effects concerning the Relationship between Family Type, Parental Depressive Symptoms, Adolescent Gender and Age, and Interaction Effects on Adolescent Problem Behavior and Psychosocial Outcomes.

Parameters	Internalizing	Externalizing	Child report of	Grade point	School	School-related
	behavior	behavior	stress	average (GPA)	problems	self-esteem
*Random effects in the empty model*						
Intercept	15.65***	3.88	9.43*	.15**	1.01	.76
Residual	40.72***	22.37***	44.78***	.50***	7.80***	27.15***
Variance by family membership	27.76%	14.78%	17.40%	23.08%	11.46%	2.72%
*Fixed effects in conditional model (including family type only)*						
Intercept	9.29***	7.75***	34.43***	7.04***	3.10***	32.39***
Family type 1 (CMC)	1.14*	−.07	.79	−.16**	.08	−.56
Family type 2 (single)	1.46**	.60	1.09*	−.14**	.37*	−.67*
*Fixed effects in the full conditional model (including family type, parental depressive symptoms, and adolescent gender and age)*						
Intercept	9.31***	7.77***	34.47***	7.04***	3.11***	32.38***
Family type 1 (CMC)	.71	−.37	−.02	−.16**	−.01	−.41
Family type 2 (single)	1.26**	.46	.72	−.13**	.31 ^b^	−.59
Parental depressive symptoms (BDI)	.81	.59	1.56***	.02	.16	−.28
Adolescent gender	2.00***	−.21	1.85***	.05	.01	−.62*
Adolescent age	1.03**	.79**	1.68***	−.13**	.48**	−.45
*Fixed effects of the full conditional model including interaction effects (best fitting model)*						
Intercept	9.00***	7.73	34.46***	7.03***	3.10***	32.46***
Family type 1 (CMC)	.57	-	-	−.17**	-	
Family type 2 (single)	1.32**	.59*	-	−.14**	-	−.32
Parental depressive symptoms (BDI)	.73	-	1.45**	.02	-	−.43
Adolescent gender	1.99***	-	1.84***	-	-	−.65*
Adolescent age	1.06**	.76**	1.73***	−.15***	.49***	
CMC*BDI	.92*	-	-	-	-	
CMC*gender	-	-	-	-	-	
CMC*age	-	-	-	.18**	-	
Single*BDI	-	-	-	-	-	.76**
Single*gender	-	-	-	-	-	
Single*age	-	-	-	.10*	-	
BDI*Gender		-	-	-	-	
BDI*Age		-	-	−.14**	-	
Gender*age	1.08**	-	.74*	-	-	

*Note*. Family type 1 =  families with a chronically ill parent. Family type 2 =  single parent families. BDI =  Beck Depression Inventory. The coefficients represent the change in effect size while controlling for the respective independent variable. The value of the coefficient needs to be added to (when positive) or subtracted from (when negative) the value of the intercept. So, for internalizing behavior, the regression coefficient of 1.14 means that compared to children in two-parent families with healthy parents, children from families with a chronically ill parent show an increase of 1.14 on their score on internalizing behavior. For children from families with single parents, this increase is 1.46.

*p≤.05.

**p<.01.

***p<.001.

### Conditional model including family type

Adolescents with chronically ill or single parents displayed more internalizing problems than controls, see Table 3. Adolescents of single parent families had significantly elevated stress scores in comparison with controls. Both target groups reported a lower GPA than the comparison group. Adolescents of single parent families reported more school problems and scored lower on school-related self-esteem than controls. Family type was not related to externalizing problems in the conditional model.

### Full conditional model

In the full conditional model, adolescents with parental CMC did no longer show significantly more internalizing problems than controls, but adolescents from single parent families still did. The target groups had a lower GPA than controls. The effect of single parent family type on child report of stress, school problems, and school-related self-esteem was no longer significant. Parental depressive symptoms showed a strong positive relationship with child report of stress. Further, being a girl and being older significantly contributed to internalizing problem behavior and stress. Older children displayed more externalizing problems, lower school grades, and more school problems than younger children.

### Best fitting model including interaction effects

Adding interaction effects to the full model and removing non-significant effects yielded four main effects of family type, see Table 3. Belonging to a single parent family predicted higher scores on internalizing and externalizing problems. Both target groups still predicted a significantly lower GPA. Girls and older children still manifested more internalizing problems and stress. As in the previous model, older children had more externalizing problems, a lower GPA, and more school problems. The main effect of parental depressive symptoms on children's stress remained significant. Four of the significant interaction effects concerned family type and the covariates. Adolescents with parental CMC and more depressive symptoms of parents had more internalizing problems than other children. Older children from the target groups had a comparatively lower GPA. Adolescents from families of healthy parents, who had lower depression scores, had a higher self-esteem compared to other children. Interactions between adolescents' gender and age, and parental depressive symptoms were only related to two adolescent outcomes. Older girls had more internalizing problems and stress than younger boys.

Exploratively, we examined whether family income was a significant covariate of adolescent outcomes, but this was not the case. We also explored effects of maternal depressive symptoms by only using maternal depressive symptoms scores instead of dyadic scores, but we did not find differences with the results presented here for the mean of maternal and paternal depressive symptoms. In addition, we calculated whether the mean score of internalizing problems or externalizing problems differed between children aged 10, 19, or 20 years and other children. Results of multilevel modeling showed that there was no difference in either internalizing problem behavior (*p* = .22) or externalizing problem behavior (*p* = .87).

### Differential outcomes between adolescents of families with a chronically ill parent and single parent families

Responding to the problem of multiple comparisons, we selected cases from families with a chronically ill parent and single parent families to make a comparison between the two target groups. Thus, families with two healthy parents were excluded from this data analysis. Adolescents with a chronically ill parent displayed significantly less externalizing problems (estimate  = −.68, *p* = .03) than adolescents of single parents. Adolescents of families with a chronically ill parent and single parent families did not differ on any other outcome variable.

## Discussion

The aim of this study was to investigate the unique and combined influence of family type and parental depressive symptoms on adolescents by comparing adolescent functioning between two-parent families with parental CMC, single parent families, and two-parent families without parental CMC (comparison group). As expected, the target groups showed a lower grade point average (GPA) than controls. Also, adolescents from single parent families showed more internalizing and externalizing problems than other children. It should be noted that most effects of family type were not consistent concerning the various outcomes and models. Most of the significant effects were related to children's gender and age and not to family type, which is in line with prior research [38].

As adolescents from single parent families reported the most elevated problem behavior scores of the three family types, they seemed to be especially vulnerable to adverse outcomes. In support of this, most research found that single parenthood was related to lower adolescent functioning, including externalizing problems [16], [18], [20], [22]. On the contrary, a meta-analysis revealed that adolescents with parental CMC do not display more externalizing problems than other children [5]. The same meta-analysis found that low socio-economic status as defined by low family income is associated with elevated children's problem behavior. It should be noted that single parent families had less income than other families. However, exploratory analyses revealed that family income did not affect adolescent outcomes significantly, and this finding was independent of family type. It is possible that adverse outcomes in our sample of adolescents of single parents emerged because of elevated parenting stress in this group [39], but this assumption reaches beyond the scope of our study.

Parental depressive symptoms seemed particularly associated with child report of stress, supporting previous research findings [2], [40]–[42]. Although parental depressive symptoms did not appear to be related to adolescent internalizing and externalizing problem behavior, it is plausible that the child's perception of stress is mediating the relationship between parental depressive symptoms and problem behavior [43]. Future research needs to address these complex mediating processes more thoroughly.

In comparison with boys, girls showed more internalizing problems, more stress, and lower self-esteem related to their academic functioning, mimicking our expectations based on prior research [44]–[46]. We did not replicate the finding that boys in the general population displayed more externalizing problems than girls [44], [45], [47]. It may be suggested that parental depressive symptoms and/or family type appears to affect girls more than boys [42].

Concerning adolescent age, we found that younger adolescents scored lower on problem behavior, supporting the notion that adolescents face more challenges with rising age, specifically within adolescence [48]. Interestingly, stress was elevated in older adolescents. Previous research did not find age to be related to stress [49], but this may be due to a power issue because of the small sample size used in the study from 2010. Also, older children reported more school problems and a lower GPA than younger children. With respect to interaction effects, it was evident that parental depressive symptoms were elevated in families with a chronically parent. Accordingly, adolescents of chronically ill parents who were also exposed to elevated parental depression were more prone to develop internalizing problems. This is an important finding, confirming our hypothesis about a potentiated effect of family type and parental depressive symptoms on adolescent functioning. Especially for adolescents with a chronically ill parent, the Screening Instrument for Adolescents with a Chronically Ill Parent (SIAPCMC) was developed [30], an instrument determining the risk for future internalizing problems. Implementing the SIAPCMC in school, care and nursing environments may contribute to the prevention of problem behavior in this particular group. Further, older children from the target groups had a comparatively lower GPA. Last but not least, children's gender and age showed a significant interaction effect on child report of stress, suggesting that older girls are especially vulnerable to stress. To conclude, when studying adolescent functioning in various family types, interaction effects with parental depression and children's demographics should be considered, too. However, more (longitudinal) research is needed to shed light on how age effects interplay with family type and parental depressive symptoms on adolescent outcomes.

Our study had some characteristics that limit conclusions concerning generalizability. Overall, the sample of participants was exclusive considering that most parents had a high income and were highly educated. Specifically adolescents with parental CMC may have been selective because they were contacted by way of medical settings and may have functioned better than an average child living in a family with parental CMC because of the availability of medical help and/or support from patient organizations. Also, the sample of CMC's was highly heterogeneous. One may argue that adolescent functioning depends on illness type, but we previously confirmed that illness type did not influence adolescent outcomes [50]. In addition to the heterogeneity of CMC's, there may have been other confounding factors affecting the conclusions, for instance, history of depressive symptoms, child rearing abilities, family conflict, and other psychosocial factors that could have influenced adolescent outcomes. Moreover, it needs to be addressed that the YSR is validated for children between 11 and 18 years of age. While the manual of the YSR for the Dutch population states that the YSR has also been administered to 10-year old and 19-year old children, these children were not included in the Dutch normative sample. We intended to cover a wide age range to be able to make statements about a larger group of adolescents, living at home with a parent with CMC or with a single parent, so we included 36 children (9% of the total sample) who were 10, 19, or 20 years of age. As in the study of Sieh et al. [51], this subsample showed good and satisfactory reliability for internalizing and externalizing problem behavior, respectively, and did not significantly differ in problem behavior scores from the remaining sample. Another limitation may be that we did not administer a clinical interview to determine depressive symptoms. Results will likely be different if a clinical diagnosis of depressive episode is applicable, but this is beyond the scope of our paper. Further, the use of self-report may be a shortcoming, because adolescents may not report their problems accurately, however, this would apply to all the three family types. In future studies hetero-reported problems and school-reported grade point average should be examined as well. Last but not least, we did not take biological, genetic or neurophysiological factors of the participants into account.

Despite several limitations, our study is strong because it examined a large sample and it is the first of its kind to examine the unique and combined effect of parental depressive symptoms on behavioral and psychosocial outcomes in adolescents from three family types, using a multilevel design. Adopting a family type perspective, this study shows that a large part of the variance in adolescent functioning can be explained by main effects of family type and parental depressive symptoms. An important interaction effect of family type and parental depressive symptoms on adolescent functioning was found, suggesting that adolescents of chronically ill parents who are highly depressed are especially vulnerable to develop internalizing problems. Adolescents of single parents comparatively displayed the lowest functioning, so it might be concluded that having a parent with CMC in two-parent families may have less impact on adolescent functioning than having a single parent. Female gender and older age, especially in this combination, proved to be the strongest predictors of adolescents' internalizing problems and stress. Considering the importance of emotional well-being and academic achievement for children's future development, we recommend professionals to be aware that children with certain family and demographic characteristics are at increased risk for problem behavior and worse psychosocial outcomes.
