# Correction: The Relationship between Parental Depressive Symptoms, Family Type, and Adolescent Functioning

**DOI:** 10.1371/annotation/b8370232-c144-4c14-8168-8dceede5b8e2

**Published:** 2013-12-10

**Authors:** Dominik Sebastian Sieh, Johanna Maria Augusta Visser-Meily, Anne Marie Meijer

The name of the first author was spelled incorrectly. The correct name is: Dominik Sebastian Sieh.

